# 1811. Impact of a one-time oritavancin infusion on hospital length of stay for uncomplicated skin and soft tissue infections

**DOI:** 10.1093/ofid/ofad500.1640

**Published:** 2023-11-27

**Authors:** Derek N Bremmer, Gabriela Alvarez, Carley Buchanan, Dustin R Carr, Nathan R Shively, Matthew A Moffa, Tamara Trienski, Thomas L Walsh

**Affiliations:** Allegheny General Hospital, Pittsburgh, Pennsylvania; Allegheny Health Network, Pittsburgh, Pennsylvania; Allegheny Health Network, Pittsburgh, Pennsylvania; Allegheny General Hospital, Pittsburgh, Pennsylvania; Allegheny Health Network, Pittsburgh, Pennsylvania; Allegheny Health Network, Pittsburgh, Pennsylvania; Allegheny General Hospital, Pittsburgh, Pennsylvania; Allegheny Health Network, Pittsburgh, Pennsylvania

## Abstract

**Background:**

Oritavancin is a lipoglycopeptide antibiotic approved in the United States for the treatment of adults with skin and soft tissue infections (SSTIs). Given the extended half-life of this agent, a one-time intravenous (IV) infusion is adequate to treat SSTIs. This single IV dose provides a convenient outpatient alternative to a traditionally dosed inpatient vancomycin regimen, which may cause a prolonged hospital length of stay (LOS). Previous studies have demonstrated oritavancin to be non-inferior to vancomycin in clinical measures of SSTI resolution. Our antimicrobial stewardship program proactively identified oritavancin eligibility to potentially decrease hospital LOS for patients with SSTIs admitted to the hospital when the primary team preferred continued IV therapy.

**Methods:**

This was a retrospective, cohort study of adult patients with uncomplicated SSTI admitted to the hospital. All patients who received oritavancin that met these criteria were included and a comparator standard of care arm (SOC) was matched based on age, Charlson score, SOFA score, location of cellulitis, and organism if available. The primary outcome was hospital LOS. Secondary outcomes included documented adverse drug events (ADEs) and 30-day emergency department (ED) visit, hospital readmission, and a composite re-evaluation in outpatient setting, ED, or hospital for continued or worsening infection.

**Results:**

A total of 57 oritavancin patients were included with 35 able to be matched to a SOC comparator. The only difference in baseline demographics was patients in the oritavancin arm were more likely to have failed outpatient antibiotics (49% vs 26%; p = 0. 048). There was no difference in hospital LOS between the oritavancin arm and SOC (2.02 vs 2.56; p = 0.250). There were similar rates of ADEs (2.8% vs 0%; p = 1.00), 30-day ED visits (11.4% vs 14.3%; p = 1.00), hospital readmissions (11.4% vs 5.7%; p = 0.673), and composite re-evaluation for continued or worsening infection (8.6% vs 17.1%; p = 0.477) in the oritavancin arm compared to SOC, respectively.

**Conclusion:**

This analysis found that patients given oritavancin for SSTIs had a numerically shorter hospital LOS, but was underpowered to detect significance. Oritavancin is a reasonable alternative when oral therapy is not an option.

**Disclosures:**

**Derek N. Bremmer, PharmD, BCIDP**, Thermo Fisher Scientific: Advisor/Consultant **Dustin R. Carr, PharmD, BCPS, BCIDP**, Merck: Advisor/Consultant **Thomas L. Walsh, MD**, Accelerate Diagnostics: Advisor/Consultant

